# Unique cutaneous metastasis of multiple myeloma

**DOI:** 10.1016/j.jdcr.2023.12.024

**Published:** 2024-03-16

**Authors:** Julien Bourgeois, Sara Charles, Elaine Huang, William Rothwell

**Affiliations:** a Department of Internal Medicine, Creighton University School of Medicine - Phoenix, Phoenix, Arizona; b Department of Internal Medicine, Tulane University School of Medicine, New Orleans, Louisiana

## Introduction

Multiple myeloma (MM) is a hematologic malignancy involving neoplastic proliferation of plasma cells producing monoclonal immunoglobulins.^1^ It is the second most common hematologic malignancy after lymphoma, with an incidence of 5 cases per 100,000.^2^ Most patients present with anemia, osteolytic bone disease, and renal failure.^3^ Cutaneous metastasis of MM is extremely rare, occurring in less than 1% of cases, often appearing late in the disease course, and associated with heavy tumor burden.^4^^,^^5^ Limited case reports describe diverse phenotypes, and the diagnosis requires histopathologic confirmation.^5^^,^^6^

We present a case of a patient with a long-standing history of MM with repeated relapses and remissions who developed a unique ulcerative rash, coalescing on the groin, thighs, and sacrum. This is a uncommon case of MM documenting its cutaneous manifestations and clinical course.

## Case presentation

Our patient is a 76-year-old woman who presented from a long-term acute care hospital with acute renal failure, melena, altered mental status, and syncope. She was first diagnosed with MM in 2005. She underwent an autologous stem cell transplant in 2006 and achieved remission of her disease until March of 2015. She was reinitiated on chemotherapy and achieved remission in January of 2022, with bone marrow biopsy showing no M spike and a K/L ratio of 13.22. She was thought to be in remission until presentation to the hospital 4 months later.

**Fig 1:**
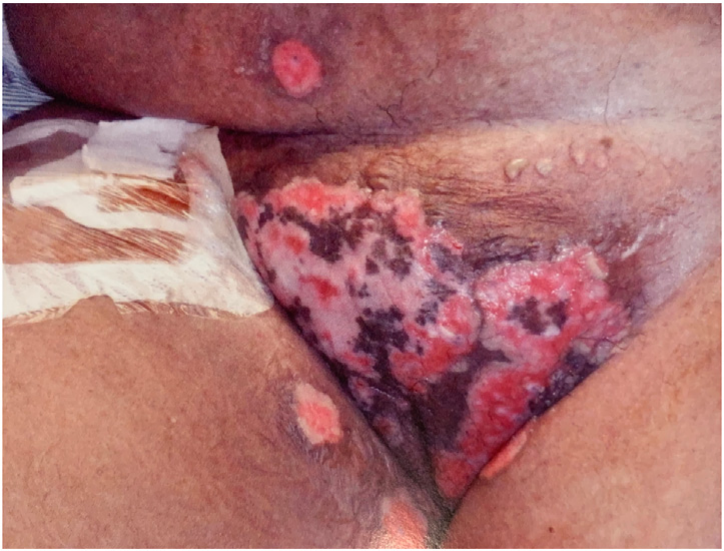
Multiple coalescing plaques with maceration and granulation tissue, few isolated erosions, and scattered early vesicular lesions with surrounding erythema and inflammatory pigment change located on the mons pubis and inner thighs.

**Fig 2:**
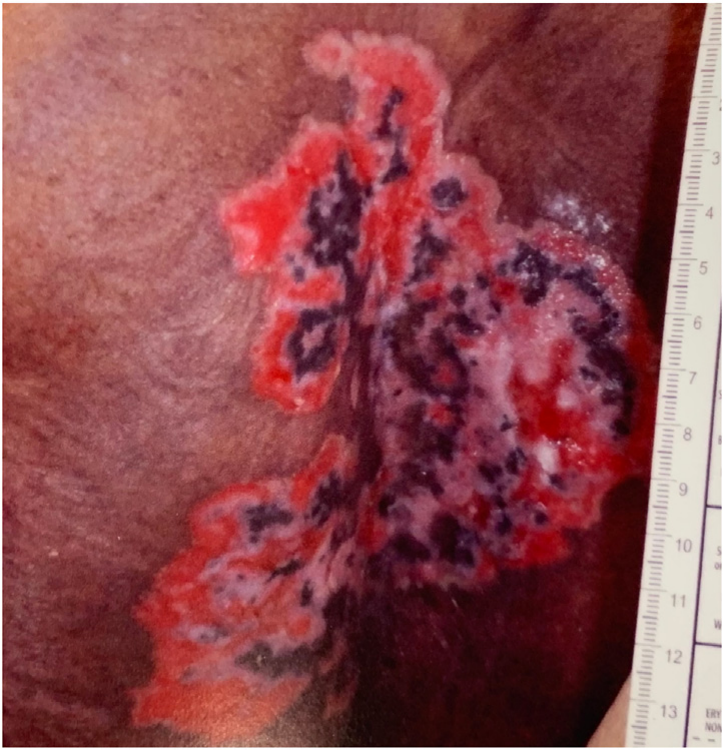
Large coalescent polymorphic erosion with areas of granulation and ulceration located on the sacrum and buttocks.

**Fig 3:**
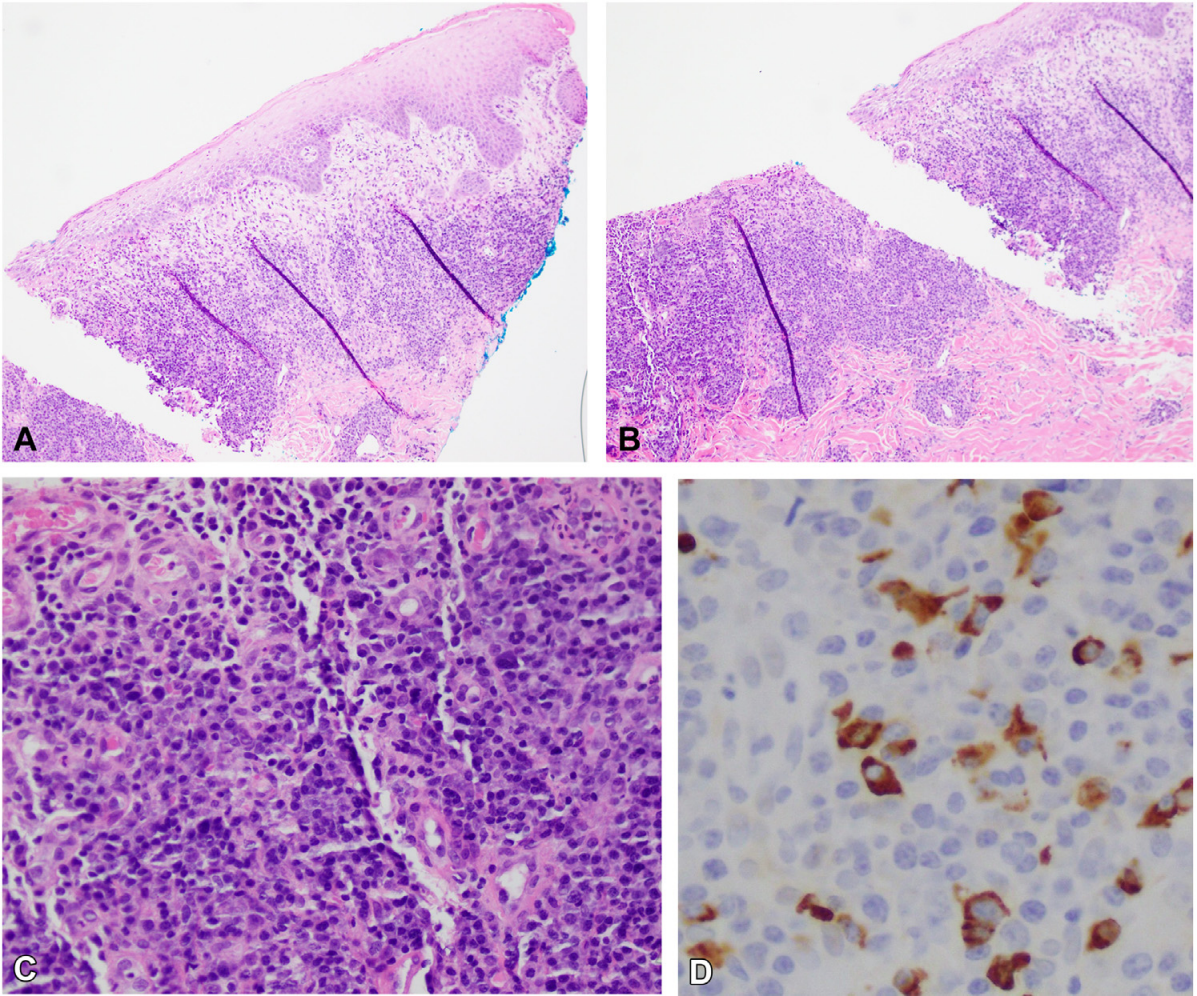
**A,** Low-power punch biopsy microscopy displaying dense plasmacytic infiltrate. **B,** Low-power punch biopsy microscopy displaying erosion of squamous epithelium and underlying dense plasmacytic infiltrate. **C,** High-power magnification of dense dermal, atypical, plasma cell infiltrate. **D,** Immunohistochemical analysis showing strong positive staining for Lambda light chain plasma cells.

On admission, physical exam showed scattered flaccid vesicles and extensive, well-demarcated, erythematous erosions and ulcerative, macerated plaques with granulation tissue coalescing on the pannus, mons, inner thighs, and sacrum (Figs 1 and 2). The skin lesions were biopsied, and the pathology report revealed a prominent lambda-restricted infiltrate of atypical plasma cells, underlying a cutaneous ulcer. These findings were consistent with an extraosseous manifestation of MM (Fig 3).

During this hospitalization, the patient was reinitiated on therapy with a 4-day course of dexamethasone, which resulted in marked clearance of the lesions. However, therapy had to be held due to systemic infection, and the lesions rapidly returned. These lesions were preceded by fluid-filled vesicles that would slough off and ulcerate before coalescing to form large areas of involvement. Throughout the remainder of her hospitalization, the patient remained severely ill and unable to tolerate further steroids or cancer therapy. Her wounds were managed with regular cleaning and dressing changes. After lengthy discussion with the family, it was determined that she was not a candidate for further therapy. She was transitioned to comfort care and passed a few weeks later.

## Discussion

The clinical presentations of cutaneous metastasis of MM reported in the literature are quite varied. The lesions can mimic cryoglobulinemia, bruising, amyloid deposition, and squamous cell carcinoma.^5^ Another case study noted diffuse eroded areas preceded by vesicular lesions with positive Nikolsky sign, pointing out possible confounding with autoimmune vesiculobullous disorders.^6^

Likewise, the histopathologic differential for plasmacytic infiltrate in the skin is broad, including primary extramedullary plasmacytoma, lymphomas, Castleman disease, various infections, and inflammatory conditions.^4^

In our case, the patient had vesicles and coalescing erosions and ulcerative plaques, appearing sporadically on the pannus, mons, groin, and thighs. These lesions responded quickly to a 4-day course of intravenous dexamethasone. Intense plasmacytic infiltrate with strong positive lambda-light chain staining was noted on histopathology. This finding is consistent with known cancer pathology, as prior clinicopathologic correlation studies have demonstrated that light chain restriction does not occur in reactive plasma cell-rich infiltrates.^4^ Therein, this patient’s lesions were consistent with cutaneous metastasis of MM.

Cutaneous involvement is a rare manifestation of MM. When diagnosed, cutaneous MM indicates a poor prognosis with a median survival of 8 months.^5^^,^^7^ Because of its various presentations and multiple confounders, biopsy with histopathologic analysis is indicated. This case contributes to the growing body of evidence surrounding cutaneous metastasis of MM.

## Conflicts of interest

None disclosed.
